# Benefits of exercise for children and adolescents with autism spectrum disorder: a systematic review and meta-analysis

**DOI:** 10.3389/fpsyt.2024.1462601

**Published:** 2024-10-07

**Authors:** Mingyuan Jia, Jia Zhang, Jianhua Pan, Fengting Hu, Zhipeng Zhu

**Affiliations:** ^1^ Department of Physical Education, Dong-A University, Busan, Republic of Korea; ^2^ School of Physical Education, Chongqing University, Chongqing, China; ^3^ College of Physical Education, Shanxi University, Taiyuan, Shanxi, China

**Keywords:** exercise, autism, positive effect, social skills, long-term approach

## Abstract

**Background:**

Numerous experimental studies have shown that exercise can serve as an intervention with beneficial effects on children and adolescents with autism. However, a systematic review on the specific areas affected has not been conducted.

**Methods:**

Preliminary research sources were obtained by searching four databases, and two researchers independently screened the literature that met the study criteria. The study was conducted under the guidelines of the Cochrane Handbook for Systematic Reviews of Interventions.

**Results:**

37 studies were included in the final analysis, of which 9 studies were quantitatively synthesized and 28 studies were qualitatively analyzed. Exercise interventions have positive effects on motor performance, cognitive function, individual and social relationships, behavioral problems, physical health, and brain function in children and adolescents with autism. The results of the meta-analysis indicate that exercise can effectively improve social skills [SMD=-0.53, 95%CI (-0.76, -0.3), P=0.000].

**Conclusions:**

Long-term, regular, chronic exercise is beneficial for children and adolescents with autism, particularly in the area of social skills.

**Systematic review registration:**

https://www.crd.york.ac.uk/prospero PROSPERO, identifier CRD42024554530.

## 1 Introduction

Autism Spectrum Disorder (ASD) is an extremely complex and highly prevalent neurodevelopmental disorder (1). Children with autism exhibit delays in communication and social skills, and demonstrate repetitive behavior patterns (2). Currently, ASD affects approximately 1% of the global population (3). Moreover, individuals with ASD face additional medical and psychiatric risks (4), such as sleep disorders (5) and executive function deficits (6).

To date, no medication can cure ASD. Conventional treatment methods, including education, psychological support therapy, and medication (7), aim to stimulate cognitive and language development while attempting to mitigate maladaptive behaviors such as stereotypy (8). These treatments also address secondary symptoms like hyperactivity, emotional instability, and aggression. Research has shown that standard treatments can have positive effects on the core issues of ASD (9). Cognitive Behavioral Therapy (CBT) can alleviate anxiety in children with ASD (10). Selective Serotonin Reuptake Inhibitors (SSRIs), commonly used to treat mood and irritability issues in ASD patients, promote neurogenesis and neuroprotection, offering potential benefits (11). However, some atypical antipsychotics have side effects, including fatigue, gastrointestinal symptoms, and more severe issues like dyslipidemia and hyperglycemia (12). Exercise, as a non-pharmacological adjunct therapy, has gained increasing attention due to its low cost and ease of implementation.

It is well known that exercise is feasible and effective in improving or assisting in the treatment of many diseases. Studies have indicated that exercise benefits social interaction deficits (13) and can reduce stereotyped behaviors in children and adolescents with autism (14, 15). Additionally, research has shown that exercise positively impacts the cognitive function of typically developing children and adolescents and reduces the potential risk of cognitive decline (16). Meanwhile, a recent meta-analysis demonstrated that exercise interventions positively affect executive functions in children and adolescents with ASD (1). Furthermore, there is evidence that social skills in ASD are related to motor skill deficits (17, 18). However, it remains inconclusive whether exercise interventions can effectively improve communication skills.

Although previous studies have systematically reviewed the effects of exercise on ASD, there has been no systematic summary specific to the population of children and adolescents. Therefore, the primary aim of this study is to systematically review the benefits of exercise for children and adolescents with ASD and to conduct a meta-analysis on the impact of exercise on social skills, in order to provide evidence-based recommendations for patient treatment.

## 2 Materials and methods

Our study was pre-registered with PROSPERO, registration number CRD42024554530.

### 2.1 Search strategy

As of April 1, 2024, a total of four databases were searched: PubMed, EMBASE, Cochrane Central Register of Controlled Trials, and Web of Science. The search strategy was designed around the study theme, combining professional terminology and keywords. The search strategy was constructed using PICO: Population: people with ASD; Intervention: exercise; Comparator: control group with usual care or no intervention; Outcomes: outcome measures for ASD. To retain all valuable studies, there were no restrictions on study time, language, or age. Detailed search strategies can be found in **Supplementary Table 1**.

### 2.2 Inclusion criteria and exclusion criteria

Participants in the studies had to be diagnosed with autism either through autism scales or by a physician. Those with a potential for autism without a confirmed diagnosis were excluded. Our study focused solely on children and adolescents, thus the upper age was limited to 18 years. The intervention for the experimental group involved exercise; however, complex exercise interventions incorporating cognitive tasks were excluded.

All studies meeting the inclusion criteria were included in the qualitative synthesis. Additionally, quantitative synthesis was conducted for the included randomized controlled trials (RCTs) that reported sufficient experimental details and required data. Observational studies, review studies, conference papers, and animal studies were excluded.

### 2.3 Study selection

Two researchers (MY and JZ) independently screened and excluded the retrieved literature using the reference manager EndNote. A third researcher (JH) reviewed and confirmed the results. The literature screening process involved excluding duplicate studies, reading titles and abstracts for further exclusion, and performing full-text reviews of the remaining articles. During the independent evaluation by the researchers, any discrepancies were resolved through discussion within the research team.

### 2.4 Data extraction

Two researchers (MY and JZ) extracted data from studies included in the quantitative synthesis. If the studies reported the required means and standard deviations, these were directly used for statistical analysis. If the required data were presented in graphical form, the data were extracted using digital science software (Engauge Digitizer). Some experiments included multiple or long-term follow-ups after the intervention and reported experimental data. To verify the intervention effects, only the data at the end of the experiment were extracted. The data extraction results were confirmed by a third researcher (JH), and any inconsistencies were resolved through team discussions. Additionally, we recorded the details of the included qualitative studies using standardized data tables.

### 2.5 Quality assessment

Two researchers (MY and JZ) conducted a methodological quality assessment of the studies included in the quantitative synthesis. The assessment tool used was the Physiotherapy Evidence Database (PEDro) scale. The PEDro scale is considered an effective tool for assessing the quality of randomized controlled trials (19). Although this scale was developed for physical therapy, it may also be suitable for other fields (20). Furthermore, existing evidence suggests that the reliability of the total score of PEDro is acceptable (21).It includes 11 criteria, such as randomization and blinding (21). However, many exercise intervention trials cannot achieve blinding, so considering this limitation, the scoring system was categorized into three quality levels: high quality (≥6 points), moderate quality (4-5 points), and low quality (≤3 points) (22). Any discrepancies in scoring between the two assessors were resolved through team discussion.

### 2.6 Data analysis

Various tools were used to measure data across different studies; therefore, the effect size needed to be calculated using the standardized mean difference (SMD), with a 95% confidence interval (CI) between groups. The effect size was interpreted as small (≤0.2), medium (≤0.5), and large (≥0.8) (23). We used the Cochrane Q test to assess statistical heterogeneity, along with I² and P-value tests. The degree of heterogeneity was classified as low (≤25%), moderate (≤50%), and high (≥75%) (24). A fixed-effect model was used for low heterogeneity, and a random-effects model was used otherwise.

## 3 Results

### 3.1 Study selection

**Figure 1:**
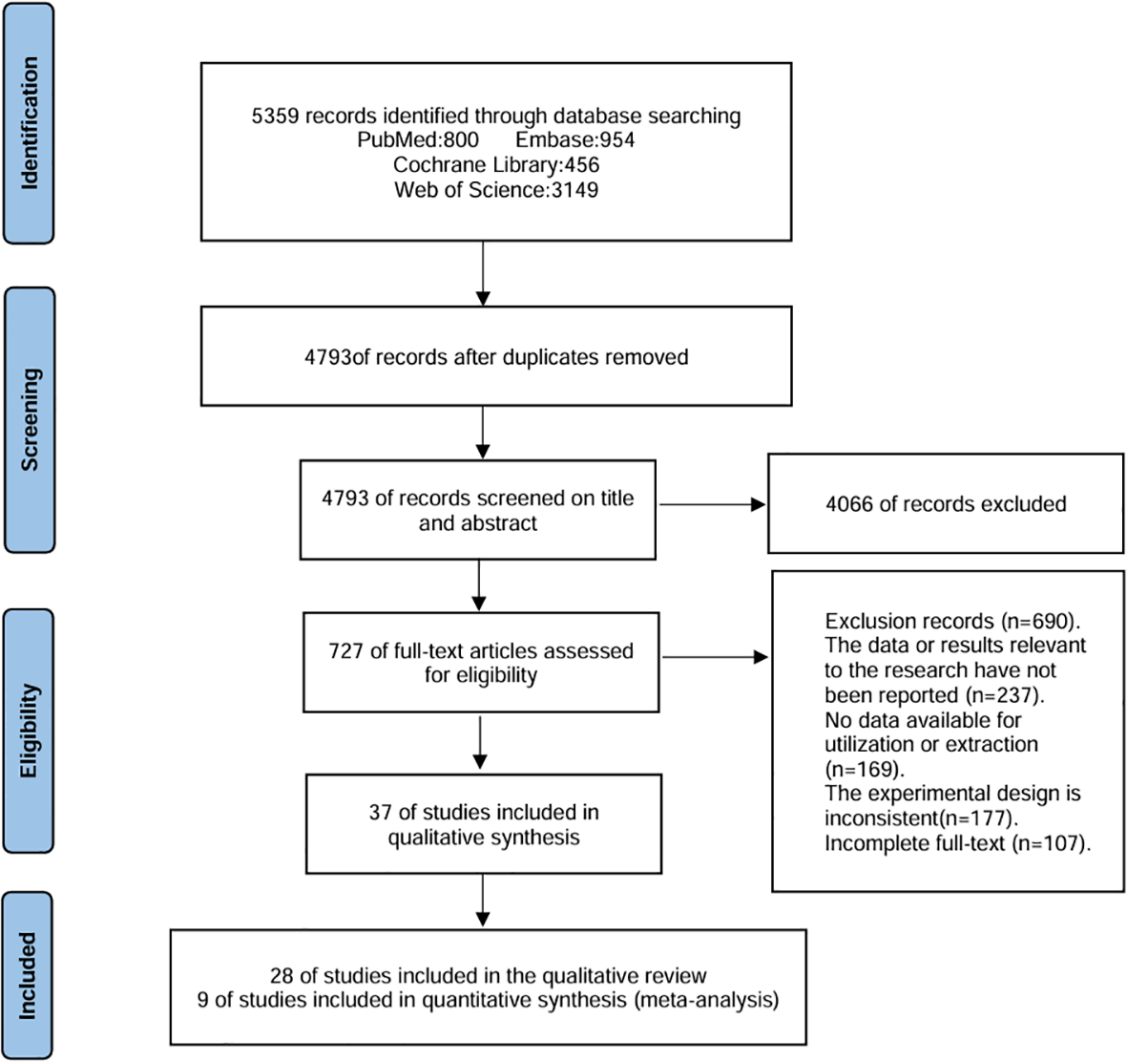
Search Flowchart.

A total of 5359 articles were identified through the initial search. After excluding duplicate studies, screening for relevant topics, and reading abstracts and full texts, 37 articles were found to meet the study criteria. Among these, 28 studies were included in the qualitative analysis (7, 25–51), and 9 studies (52–60) were included in the meta-analysis. The detailed selection process is illustrated in **Figure 1**.

### 3.2 Study characteristics

**Table 1** presents the detailed information of the included studies. The country with the most studies was Iran, with a total of 9 studies. China conducted 8 studies, the United States had 6 studies, India had 3 studies, Brazil, Turkey, and Australia each had 2 studies, and the UK, Tunisia, Switzerland, Italy, and Portugal each had 1 study. The age range of the 1553 participants was 2.3-17.3 years, with a higher number of male children and adolescents compared to females. The methods of symptom diagnosis included physician diagnosis, ICD-10, ADOS-2, DSM-4, DSM-5, and GARS2 scale, but 4 studies did not report specific diagnostic methods.

**Table 1:** Detailed information on qualitative analysis.

Author, Country, Design Type	Participant characteristics			Content of the experimental intervention					
	Age	Number of people	Diagnostic methods/ scales	Methods of intervention (Control Group)	Type of exercise (intensity of exercise)	Total length of intervention	Intervention frequency	Duration of intervention	Outcome Indicators Data with significant results (p-value)
**Morteza Homayounnia Firouzjah; Iran; PTPT**	9-11	M:30	Medical diagnosis	SMIE (NI)	Chronic	48 weeks	3 times a week	45min	Social Skills (P< 0.01); Motor performance (P< 0.01)
**Chaoxin Ji; China; RCT**	VT:12.5 ± 2.36; PE:13.1 ± 2.97; CG:12.8 ± 2.69	M:55 F:45	ICD-10	PE/VT (PSY)	Chronic	6 weeks	3 times a week	60mn	CRRT; DRPT; PSIR: DRPS:P< 0.05; MT:P< 0.05; MNT:P< 0.05; PSIR:P< 0.05
**Chaoxin Ji; China; RCT**	VT:12.5 ± 2.36; PE:13.1 ± 2.97; CG:12.8 ± 2.69	M:55 F:45	ICD-10	PE/VT (PSY)	Chronic (LMAE)	6 weeks	3 times a week	60mn	Executive Function (P< 0.001); Flexibility (P< 0.001); Inhibition (P< 0.001)
**Homa Rafiei Milajerdi; Iran; RCT**	SPARK: 7.95 ± 1.60; Kinect:8.15 ± 1.5; CG:8.45 ± 1.43	M:57; F:3	ADOS-2	SPARK/Kinect (NI)	Chronic (LMAE)	8 weeks	3 times a week	35min	Motor skills (ACD, P< 0.05); Executive Function (CF, P< 0.01)
**Hadi Moradi; Iran; PTPT**	7.62 ± 1.15	M:50	DSM-5	PME (PD)	Chronic	3 mnths	3 times a week	60min	Stereotypical behavior (P=0.01)
***Mengxian Zhao; China; RCT***	EG:6.14 ± 0.96; CG:6.1 ± 0.98	M:29; F:12	DSM-5	SPAP (RPA)	Chronic	12 weeks	2 times a week	60min	Social skills (P< 0.005)
***Supritha Aithal; United Kingdom; RCT***	8-13	M:21; F:5	DSM-5	DMP (SC)	Chronic	5 weeks	2 times a week	40min	Social and Emotional Wellbeing (SCQ, P=0.001)
***Agnes S. Chan; China; RCT***	EG:11.28 ± 3.9; CG:12.42 ± 3.25	M:36; F:4	DSM-4	NYG (PMR)	Chronic	4 weeks	2 times a week	60min	Self-Control (Rule Violation: P< 0.05; Completion Time: P< 0.05; Unique Designs: P< 0.05; Communication; P< 0.05); Brain Activity
**Xingda Ju; China; RCT**	EG:11.11 ± 2.52; CG:12.75 ± 2.31	M:12; F:5	Medical diagnosis	Yoga (NI)	Chronic	8 weeks	3 times a week	45-50min	Problem Behavior: (Irritability, Social Withdrawal, P< 0.05); Motor Coordination (Ball Skills (P< 0.001); Static and Dynamic Balance, P=0.028)
**Maninderjit Kaur; USA; PTPT**	5-13	M:22; F:2	ADOS-2	Yoga (Academic)	Chronic	8 weeks	4 times a week	20-45min	Motor Proficiency (gross motor,P< 0.006); Training-specific Imitation (P< 0.02)
**Mostafa Sarabzadeh; Iran; RCT**	EG:8.88 ± 1.76; CG:8.22 ± 1.92	M:14; F:4	GARS2	Tai Chi Chuan (NI)	Chronic	6 weeks	3 times a week	60min	Motor skills (Ball skills, P< 0.05; Balance performance, P< 0.05)
**Sindhu Shanker; India; RCT**	EG:9.77 ± 2.63; CG:9.61 ± 1.93	M:35; F:8	Medical diagnosis	Group yoga (NI)	Chronic	12 weeks	NR	45min	Motor Proficiency (Brieftotal,P=0.005; Manual coordination,P=0.019; Body coordination,P=0.01)
***Sindhu Shanker; India; RCT***	EG:9.77 ± 2.63; CG:9.61 ± 1.93	M:35; F:8	Medical diagnosis	Group yoga (NI)	Chronic	12 weeks	NR	45min	Social Responsiveness (Social communication,P=0.021; Social Cognition,P=0.008; Social Cognition); Social Motivation subscale,P=0.042; RRB:P=0.016); Problem behaviors (Irritability,P=0.041; Social withdrawal,P=0.047)
***Mohammad Saber Sotoodeh; Iran; RCT***	EG:10.8 ± 2.36; CG:10.5 ± 1.87	M; 21; F:8	DSM-5	Yoga (NI)	Chronic	8 weeks	3 times a week	30min	Sociability (P=0.001); Sensory/Cognitive/Awareness (P=0.004); Health/Physical/Behavior (P=0.001);
***Lavinia Teixeira-Machado; Brazil; RCT***	EG:10.412.24; CG:10 ± 2.03	M:29; F:7	DSM-5	Dance (SC)	Chronic	24 weeks	1 time a week	40min	Communication (P< 0.001); Social cognition (P< 0.001); Autistic behavior (P< 0.001)
**Soleyman Ansari; Iran; RCT**	8-14	M:30	DSM-5	Aquatic exercise/karate (NI)	Chronic	10 weeks	2 times a week	60min	Static balance (P< 0.05); Dynamic balance (P=0.001)
**Hamza Marzouki; Tunisia; RCT**	6-7	M:21; F:7	DSM-5	Aquatic exercise (NI)	Chronic	8 weeks	2 times a week	50min	Gross motor skills (Locomotor’ s ability, P< 0.001; Control skills, P< 0.001); Stereotypy behavior (P=0.014); Emotion Regulation
**Fahimeh Hassani; Iran; RCT**	8-11	M:20; F:10	DSM-5	SPARK/ICPL (NI)	Chronic	8 weeks	2 times a week	60min	Gross skill (P=0.005); Fine skill (P=0.005); Motor skill (P=0.005)
***Fatimah Bahrami; USA; RCT***	EG:9.2 ± 3.32; CG:9.06 ± 3.33	M:26; F:4	DSM-4	KTT (Education)	Chronic	14 weeks	4 times a week	30-90min	Communication deficit (P=0.000)
***Janice N. Phung; USA; RCT***	EG:9.1 ± 1.1; CG:9.52 ± 1.07	M:28; F:6	ADOS-2	MMAT (NI)	Chronic	13 weeks	2 times a week	45min	Social skills (P< 0.01); Problem behaviors (P< 0.01)
***Kelong Cai; China; RCT***	EG:5.13 ± 0.61; CG:4.68 ± 0.72	M:25; F:4	DSM-5	MBT (NI)	Chronic (MHE)	12 weeks	5 times a week	40min	Social communication (P< 0.05); Ssocial cognition (P< 0.05); White Matter Structure
**Emine Adin; Turkey; PTPT**	EG:10.25 ± 0.46; CG:10.57 ± 1.27	M:13; F:2	NR	Swimming (NI)	Chronic	6 weeks	3 times a week	60min	FEV (P=0.017); FVC (P=0.03); MVV (P=0.017); IVC (P=0.025)
**Mahrokh Dehghani; Iran; PTPT**	EG:9.2 ± 0.6; CG:9.4 ± 0.5	M:24	GARS2	SPARK (NI)	Chronic	6 weeks	3 times a week	45min	PVGR (P=0.001); Loading rate (P=0.009); PPMHR (P=0.021)
**Brian C. Helsel; USA; PTPT**	13.2 ± 2.2	M:11; F:8	Medical diagnosis	Yoga	Chronic	6 weeks	3 times a week	30min	Physical activity-related skills (Leg strength, P< 0.05; Flexibility, P=0.008; Dynamic balance, P=0.001)
**Katherine Howells; Australia; PTPT**	EG:8.01 ± 1.8; CG:8.73 ± 2.33	M:32; F:3	DSM-5/ ICD-10	CBFP (NR)	Chronic	10-22 weeks	1 time a week	60-90min	Total motor ability (P=0.03); Aiming and catching (P< 0.001); Balance (P=0.01); Manual dexterity
**Katherine Howells; Australia; PTPT**	EG:7.98 ± 1.71; CG:8.62 ± 2.26	M:37; F:3	DSM-4/5	CBFP (NI)	Chronic	4-21 weeks	1 time a week	60-90min	Behavior problems (Total, P=0.048; Internalizing, P=0.01; Anxiety, P=0.001; Social problems, P=0.01)
**Maninderjit Kaur; USA; PTPT**	5-13	M:21; F:2	ADOS-2	Yoga (Academic)	Chronic	8 weeks	4 times a week	45min	JAT (P< 0.05); SDVC (P=0.05); GIA (P< 0.02); LNA (P< 0.02)
**Sebastian Ludyga; Switzerland:**	EG:10.9 ± 1.5; CG:10.4 ± 2.0	M:29; F:1	DSM-5	Cycling (Watch video)	Acute (MI)		1 time	20min	Cognitive performance (Increased reaction time, P=0.044); HNN (P< 0.001); GCPS (P=0.049)
**Emanuela Pierantozzi; Italy; PTPT**	EG:11.1 ± 1.9; CG:11 ± 1.5	40	DSM-5	Judo Program (NI)	Chronic	6 months	1 time a week	90min	Cardiorespiratory fitness, (VO2max,P< 0.05); Waist circumference (P< 0.001)
**Lucy E. Rosenblatt; USA; PTPT**	3.6-16.5	M:22; F:2	NR	Yoga & Dance	Chronic	8 weeks	1 time a week	45min	BASC-2 (BSI, P=0.04; Atypicality, P=0.02);
**Roza Tabeshian; Iran;** **RCT**	6-12	M:19; F:4	Medical diagnosis	Ta chi chuan (NR)	Chronic	12 weeks	3 times a week	45min	Stereotypic behavior (P=0.001)
**Chrystiane V. A. Toscano; Portugal: RCT**	EG:8.2 ± 1.7; CG; 8.9 ± 2.0	M:56; F:8	DSM-4	PAE (SC)	Chronic	48 weeks	2 times a week	40min	Metabolic indicators (high-density lipoprotein cholesterol, low-density lipoprotein cholesterol, and total cholesterol); Autism traits; PQL
**Chrystiane V. A. Toscano; Brazil; PTPT**	2.3-17.3	M:196; F:33	DSM-5	PAE (SC)	Chronic (MI)	48 weeks	2 times a week	30min	Social interaction problems, Attention deficit, Emotional reactivity, Stereotypical verbal, motor behavior, Asleep Disturbances (Decreased)
**HM Vidyashree; India; RCT**	EG:10.56 ± 3.98; CG:11.7 ± 4.28	M:23; F:5	NR	Yoga (SC)	Chronic	3 months	7 times a week	40min	Mean Heart rate, (Reduction, P=0.03); SDNN (Increase, P=0.04); PNN50% (Decrease, P=0.02) Effect parasympathetic dominance
**Jin-Gui Wang; China; RCT**	EG:5.11 ± 0.65; CG:4.7 ± 0.70	M; 28; F:5	DSM-5	Basketball (NI)	Chronic	12 weeks	5 times a week	40min	Working memory (P< 0.01); Regulation (P< 0.05); Social Communication (P< 0.05); Repetitive behavior (P< 0.05);
**YUSUF BURAK YAMANER; Turkey, RCT**	8-10	30	NR	Aerobic Exercises	Chronic	3 months	53times a week	45min	Coordination (P=0.001); Agility (P=0.001)
**Dongyue Zhou; China; RCT**	EG:6.4 ± 2.07; CG:5.94 ± 1.79	M:50; F:8	DSM-5	Basketball (NI)	Chronic	12 weeks	5 times a week	40min	Improve brain functional and structural networks

PTPT, pre-test-post-test; SMIE, sensory-motor integration exercises; NI, No intervention; PE, Physical exercise; VT, Virtual training; RCT, Randomized controlled trials; PSY, Psychological counseling; CG, Control group; ICD-10, International Classification of Diseases, 10th edition; DRPS, Detection rate of probe stimulus; MT, Moving target; MNT, Moving non-target; PSIR, Probe stimulus inhibition rate; CRRT, Correct rate of ring tracking; DRPT, Detection rate of probe stimulus; CRPT, Correct rate of probe stimulus; LMAE, Light to moderate aerobic exercise; WM, Working memory; ADOS-2, Autism Diagnostic Observation Schedule-Second Edition; ACD, Aiming and catching domain; CF, Cognitive flexibility; PME, Perceptual-motor exercises; PD, Placebo drops; DSM-5, Diagnostic and Statistical Manual of Mental Disorders-Fifth Edition, Text Revision; EG, Experimental group; SPAP, structured physical activity program; RPA, Regular physical activity; DMP, Dance Movement Psychotherapy; SCQ, Social communication questionnaire; NYG, Nei Yang Gong; PMR, Progressive muscle relaxation; SC, Standard care; GARS2, Gilliam Autism Rating Scale- second edition; RRB, Restricted, repetitive behaviors; ICPL, I Can have a physical literacy; KTT, Karate Techniques Training; MMAT, Mixed martial arts training; MBT, Mini-Basketball Training; MHE, Moderate to high exercise; FEV, second forced expiratory volume; , FVC, Forced vital capacity; MVV, Maximal voluntary ventilation; IVC, Inspiratory vital capacity; PVGR, The first peak of vertical ground reaction force; PPMHR, Peak pressure at the medial heel region; CBFP, Community− Based Football Program; SDVC, Socially directed verbal communication; LNA, Less negative affect; GIA, Greater interested affect; JAT, Joint Attention Test; MI, Moderate Intensity; HNN, higher negativity of N170 amplitudes; GCPS, greater constriction of pupil size; BASC-2, The Behavioral Assessment System for Children, Second Edition; BSI, Behavioral Symptom Index, PAE; Physical activity program; PQL, Parent-perceived quality of life; MI, Moderate intensity; SDNN, Standard deviation of all normal RR intervals; PNN50%, The proportion derived by dividing NN50 by the total number of NN intervals.

Five studies reported the specific intensity of exercise, with only one study focusing on acute exercise interventions, while the others focused on long-term exercise interventions. Regarding the frequency and duration of interventions, interventions were conducted at least once a week, with a maximum of 7 times per week, and the duration of each session ranged from 20 minutes to 90 minutes. Most studies reported positive effects of exercise, whereas the acute exercise intervention reported negative effects. In the assessment of social skills, the test content focuses on language, communication, and overall social skills evaluation. The judgment of experimental results mainly centers on the scores of the relevant scales, as these scores can specifically reflect whether the intervention has had a tangible impact on the patient, be it positive or negative.

### 3.3 Quality assessment

**Table 2** presents the quality assessment of the studies included in the quantitative analysis. Overall, the quality of the studies included in the meta-analysis was above average, with an overall mean score of 6.33. All studies reported the eligibility criteria for participants and achieved random allocation in the grouping. However, due to the nature of the control group not engaging in exercise, blinding of participants and therapists was not feasible.

**Table 2:** Quality assessment results.

Study	EC	RA	CA	SAB	SB	TB	AB	DR	ITA	BC	PM	TS	SQ
**Mengxian Zhao**	Y	Y	Y	Y	N	N	N	N	Y	Y	Y	6	High
**Supritha Aithal**	Y	Y	Y	Y	N	N	N	N	N	Y	Y	5	Moderate
**Agnes S. Chan**	Y	Y	Y	Y	Y	N	N	N	Y	Y	Y	7	High
**Sindhu Shanker**	Y	Y	N	Y	N	N	N	N	Y	Y	Y	5	Moderate
**Mohammad Saber Sotoodeh**	Y	Y	Y	Y	N	Y	Y	Y	Y	Y	Y	9	High
**Lavinia Teixeira-Machado**	Y	Y	Y	Y	N	N	Y	N	Y	Y	Y	7	High
**Fatimah Bahrami**	Y	Y	Y	Y	N	N	N	Y	Y	Y	Y	7	High
**Janice N. Phung**	Y	Y	Y	Y	N	N	N	N	Y	Y	Y	6	High
**Kelong Cai**	Y	Y	N	Y	N	N	N	N	Y	Y	Y	5	Moderate

EC, Eligibility criteria; RA, Random allocation; CA, Concealed allocation; SAB, Similar at baseline; SB, Subject blinded; TB, Therapist Blinded; AB, Assessor blinded; DR, Dropout rate; ITA, Intention-to-treat analysis; BC, Between-group comparison, PM, Points measures; TS, Total score; SQ, Overall study quality; Y, Yes; N, No.

### 3.4 Meta-analysis of the effect of exercise on social skills

**Figure 2:**
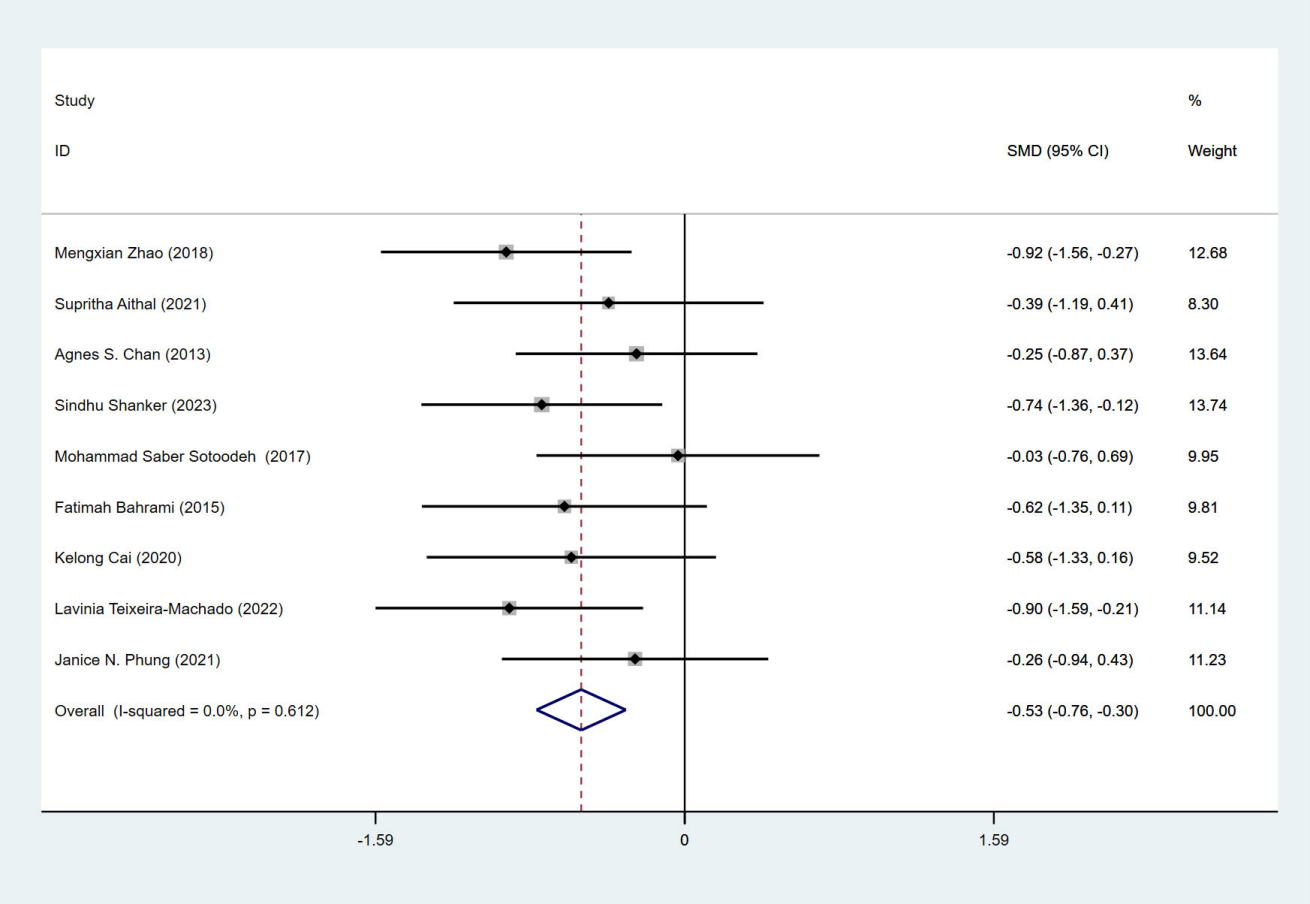
Meta-analysis forest plot on the effects of exercise on social skills.

Nine studies reported the impact of exercise on the social function of children and adolescents with autism, as shown in **Figure 2**. Compared to the control group, exercise effectively improved social skills, demonstrating a moderate effect size [SMD=-0.53, 95% CI (-0.76, -0.3), P=0.000]. The statistical heterogeneity results indicated low heterogeneity among the included studies (I²=0.0%, P=0.612).

### 3.5 Publication bias

Although the number of studies included in the meta-analysis did not exceed ten, a funnel plot was drawn to identify potential publication bias (see **Supplementary Table 2**). A visual inspection of the funnel plot showed no obvious asymmetry, and the Egger test results (P=0.328) also indicated no significant publication bias, suggesting that the study results are reasonably robust.

## 4 Discussion

We utilized a standardized literature search and screening process and determined the effect size of the meta-analysis based on the existing data. The current evidence indicates that most long-term chronic exercise interventions have positive effects on children and adolescents with autism, particularly in the area of social skills.

Overall, the positive effects of exercise primarily encompass improvements in motor performance, cognitive function, individual and social relationships, behavioral problems, physical health, and brain function. This suggests that exercise can serve as an effective intervention method. The types of interventions mainly included aerobic exercises, aquatic exercises, mind-body exercises, and ball sports. However, the variations in exercise intervention types, intensities, and doses make it difficult to determine which specific methods and intervention frequencies yield the maximum benefits. The findings highlight the importance of exercise as a beneficial intervention for children and adolescents with autism. Yet, further research is needed to identify the optimal types, intensities, and frequencies of exercise interventions to maximize the benefits for this population.

In terms of motor performance, chronic exercise has been shown to improve gross motor abilities such as motor coordination, dynamic and static balance, as well as fine motor skills. Existing research indicates that exercises like walking and strength training are effective alternative therapies for improving motor skills in children with ASD (61, 62). Similarly, there is evidence supporting the enhancement of cognitive abilities through exercise, particularly in executive functions of ASD. A meta-analysis of randomized controlled trials demonstrated significant training effects of exercise interventions on overall executive functions in children with ASD and Attention Deficit Hyperactivity Disorder (ADHD) (63). Positive effects of exercise on cognitive abilities have also been observed in other neurodevelopmental disorder populations, such as children with intellectual disabilities (64). Regarding improvements in social relationships, we speculate that these may be linked to enhancements in communication skills. Improvements in indicators such as social responsibility and reduced social withdrawal are closely associated with improved communication with others. Exercise is known to enhance physical health by improving cardiovascular function, a well-established finding (65), which holds true for individuals with ASD as well.

Furthermore, through meta-analysis, we found significant improvements in social functioning among children and adolescents with ASD following regular exercise interventions. Previous studies have reported an association between motor skills and communication abilities. There is evidence of a correlation between motor skills and adaptive social skills or adaptive communication (18). However, the potential reasons for the correlation between motor skills and social skills in patients with autism remain unclear. Thus, the underlying mechanisms may involve multiple aspects, such as neurophysiological mechanisms, behavioral and psychological mechanisms. Exercise influences levels of neurotransmitters such as dopamine (66), which play important roles in emotional regulation and behavior. Enhancing these neurotransmitter levels through exercise may improve emotional states, reduce anxiety, and thereby facilitate social interactions. In terms of behavioral and psychological mechanisms, the sense of achievement that exercise brings to children and adolescents with ASD may increase their confidence and willingness to engage in social interactions. Through interactions and correlations among these potential influencing mechanisms, overall improvements in social capabilities are promoted. Of course, there are likely many other mechanisms influencing the relationship between exercise and social skills.

This study has both strengths and limitations. Firstly, we conducted the first meta-analysis examining the effects of exercise interventions on social skills in children and adolescents with ASD. Secondly, the quantitative analysis focused exclusively on randomized controlled trials, excluding observational studies, thereby enhancing the reliability and accuracy of the study outcomes. We targeted a precise intervention for children and adolescents in developmental stages, which is advantageous. However, this study also has limitations. Firstly, the number of studies included in the quantitative analysis was limited, potentially compromising the reliability of the effects of exercise interventions and making it difficult to draw definitive conclusions. Moreover, the limited number of studies suggests that other potential moderating factors may have influenced the results. Thirdly, due to differences in variables such as the severity of ASD and intervention dosages among the included studies, subgroup comparisons were not feasible.

## 5 Conclusions

Based on the analysis of existing literature, chronic exercise interventions are beneficial for children and adolescents with ASD, particularly in improving social skills.

## Data Availability

The original contributions presented in the study are included in the article/**Supplementary Material**. Further inquiries can be directed to the corresponding author.
