# Supplementary material for: Benefits of exercise for children and adolescents with autism spectrum disorder: a systematic review and meta-analysis
Source: Front Psychiatry. 2024 Oct 7;15:1462601. doi: 10.3389/fpsyt.2024.1462601 (PMC11491325; doi:10.3389/fpsyt.2024.1462601)
Supplement: Supplementary file 1 [file Table1.docx]

Supplementary Material

**Detailed Literature Search Strategy**

**Appendix 1** Search Strategy

| Database | Search strategy | amount |
| --- | --- | --- |
| PubMed | | |
| #1 | Search: Autistic Spectrum Disorder[MeSH Terms] | 50770 |
| #2 | Search: ((((((((((((((((Autistic Spectrum Disorders[Title/Abstract]) OR (Disorder, Autistic Spectrum[Title/Abstract])) OR (Autism Spectrum Disorders[Title/Abstract])) OR (Disorder, Autistic[Title/Abstract])) OR (Disorders, Autistic[Title/Abstract])) OR (Autism[Title/Abstract])) OR (Autism, Early Infantile[Title/Abstract])) OR (Early Infantile Autism[Title/Abstract])) OR (Infantile Autism, Early[Title/Abstract])) OR (Autism, Infantile[Title/Abstract])) OR (Infantile Autism[Title/Abstract])) OR (Kanner's Syndrome[Title/Abstract])) OR (Kanners Syndrome[Title/Abstract])) OR (Kanner Syndrome[Title/Abstract])) OR (ASD[Title/Abstract])) OR (Pervasive Child Development Disorders[Title/Abstract])) OR (Pervasive Development Disorders[Title/Abstract]) | 77436 |
| #3 | Search: (#1) OR (#2) | 84576 |
| #4 | Search: Exercises[MeSH Terms] | 295002 |
| #5 | Search: (((((((((((((((((((((((Exercise, Physical[Title/Abstract]) OR (Exercises, Physical[Title/Abstract])) OR (Physical Exercise[Title/Abstract])) OR (Physical Exercises[Title/Abstract])) OR (Physical Activity[Title/Abstract])) OR (Activities, Physical[Title/Abstract])) OR (Activity, Physical[Title/Abstract])) OR (Physical Activities[Title/Abstract])) OR (Exercise, Aerobic[Title/Abstract])) OR (Aerobic Exercise[Title/Abstract])) OR (Aerobic Exercises[Title/Abstract])) OR (Exercises, Aerobic[Title/Abstract])) OR (Exercise, Isometric[Title/Abstract])) OR (Exercises, Isometric[Title/Abstract])) OR (Isometric Exercises[Title/Abstract])) OR (Isometric Exercise[Title/Abstract])) OR (Acute Exercise[Title/Abstract])) OR (Acute Exercises[Title/Abstract])) OR (Exercise, Acute[Title/Abstract])) OR (Exercises, Acute[Title/Abstract])) OR (Exercise Training[Title/Abstract])) OR (Exercise Trainings[Title/Abstract])) OR (Training, Exercise[Title/Abstract])) OR (Trainings, Exercise[Title/Abstract]) | 281873 |
| #6 | Search: (#4) OR (#5) | 446638 |
| #7 | Search: (#3) AND (#6) | 800 |

| Database | Search strategy | amount |
| --- | --- | --- |
| Embase | | |
| #1 | 'autistic spectrum disorder'/exp OR 'autistic spectrum disorder' OR (autistic AND ('spectrum'/exp OR spectrum) AND ('disorder'/exp OR disorder)) OR 'autistic spectrum disorders':ab,ti OR 'disorder, autistic spectrum':ab,ti OR 'autism spectrum disorders':ab,ti OR 'disorder, autistic':ab,ti OR 'disorders, autistic':ab,ti OR autism:ab,ti OR 'autism, early infantile':ab,ti OR 'early infantile autism':ab,ti OR 'infantile autism, early':ab,ti OR 'autism, infantile':ab,ti OR 'infantile autism':ab,ti OR 'kanners syndrome':ab,ti OR 'kanner syndrome':ab,ti OR asd:ab,ti OR 'pervasive child development disorders':ab,ti OR 'pervasive development disorders':ab,ti | 131330 |
| #2 | exercises OR 'exercise, physical':ab,ti OR 'exercises, physical':ab,ti OR 'physical exercise':ab,ti OR 'physical exercises':ab,ti OR 'physical activity':ab,ti OR 'activities, physical':ab,ti OR 'activity, physical':ab,ti OR 'physical activities':ab,ti OR 'exercise, aerobic':ab,ti OR 'aerobic exercise':ab,ti OR 'aerobic exercises':ab,ti OR 'exercises, aerobic':ab,ti OR 'exercise, isometric':ab,ti OR 'exercises, isometric':ab,ti OR 'isometric exercises':ab,ti OR 'isometric exercise':ab,ti OR 'acute exercise':ab,ti OR 'acute exercises':ab,ti OR 'exercise, acute':ab,ti OR 'exercises, acute':ab,ti OR 'exercise training':ab,ti OR 'exercise trainings':ab,ti OR 'training, exercise':ab,ti OR 'trainings, exercise':ab,ti | 341093 |
| #3 | #1 and #2 | 954 |

| Database | Search strategy | amount |
| --- | --- | --- |
| Web of Science | | |
| #1 | (((((((((((((((((TS=(Autistic Spectrum Disorder)) OR TS=(Autistic Spectrum Disorders)) OR TS=(Disorder, Autistic Spectrum)) OR TS=(Autism Spectrum Disorders)) OR TS=(Disorder, Autistic)) OR TS=(Disorders, Autistic)) OR TS=(Autism)) OR TS=(Autism, Early Infantile)) OR TS=(Early Infantile Autism)) OR TS=(Infantile Autism, Early)) OR TS=(Autism, Infantile)) OR TS=(Infantile Autism)) OR TS=(Kanner's Syndrome)) OR TS=(Kanners Syndrome)) OR TS=(Kanner Syndrome)) OR TS=(ASD)) OR TS=(Pervasive Child Development Disorders)) OR TS=(Pervasive Development Disorders) | 149997 |
| #2 | ((((((((((((((((((((((((TS=(Exercises)) OR TS=(Exercise, Physical)) OR TS=(Exercises, Physical)) OR TS=(Physical Exercise)) OR TS=(Physical Exercises)) OR TS=(Physical Activity)) OR TS=(Activities, Physical)) OR TS=(Activity, Physical)) OR TS=(Physical Activities)) OR TS=(Exercise, Aerobic)) OR TS=(Aerobic Exercise)) OR TS=(Aerobic Exercises)) OR TS=(Exercises, Aerobic)) OR TS=(Exercise, Isometric)) OR TS=(Exercises, Isometric)) OR TS=(Isometric Exercises)) OR TS=(Isometric Exercise)) OR TS=(Acute Exercise)) OR TS=(Acute Exercises)) OR TS=(Exercise, Acute)) OR TS=(Exercises, Acute)) OR TS=(Exercise Training)) OR TS=(Exercise Trainings)) OR TS=(Training, Exercise)) OR TS=(Trainings, Exercise) | 1692198 |
| #3 | #33 AND #32 | 3149 |

| Database | Search strategy | amount |
| --- | --- | --- |
| Cochrane | | |
| #1 | (Autistic Spectrum Disorder):ti,ab,kw OR (Autistic Spectrum Disorders):ti,ab,kw OR (Disorder, Autistic Spectrum):ti,ab,kw OR (Autism Spectrum Disorders):ti,ab,kw OR (Disorder, Autistic):ti,ab,kw | 4687 |
| #2 | Disorders, Autistic):ti,ab,kw OR (Autism):ti,ab,kw OR (Autism, Early Infantile):ti,ab,kw OR (Early Infantile Autism):ti,ab,kw OR (Infantile Autism, Early):ti,ab,kw | 5489 |
| #3 | (Autism, Infantile):ti,ab,kw OR (Infantile Autism):ti,ab,kw OR (Kanner's Syndrome):ti,ab,kw OR (Kanners Syndrome):ti,ab,kw OR (Kanner Syndrome):ti,ab,kw | 48 |
| #4 | (ASD):ti,ab,kw OR (Pervasive Child Development Disorders):ti,ab,kw OR (Pervasive Development Disorders):ti,ab,kw | 3426 |
| #5 | #1 or #2 or #3 or #4 | 6203 |
| #6 | Exercises):ti,ab,kw OR (Exercise, Physical):ti,ab,kw OR (Exercises, Physical):ti,ab,kw OR (Physical Exercise):ti,ab,kw OR (Physical Exercises):ti,ab,kw | 146645 |
| #7 | (Physical Activity):ti,ab,kw OR (Activities, Physical):ti,ab,kw OR (Activity, Physical):ti,ab,kw OR (Physical Activities):ti,ab,kw OR (Exercise, Aerobic):ti,ab,kw | 90819 |
| #8 | (Aerobic Exercise):ti,ab,kw OR (Aerobic Exercises):ti,ab,kw OR (Exercises, Aerobic):ti,ab,kw OR (Exercise, Isometric):ti,ab,kw OR (Exercises, Isometric):ti,ab,kw | 23730 |
| #9 | (Isometric Exercises):ti,ab,kw OR (Isometric Exercise):ti,ab,kw OR (Acute Exercise):ti,ab,kw OR (Acute Exercises):ti,ab,kw OR (Exercise, Acute):ti,ab,kw | 17860 |
| #10 | (Exercises, Acute):ti,ab,kw OR (Exercise Training):ti,ab,kw OR (Exercise Trainings):ti,ab,kw OR (Training, Exercise):ti,ab,kw OR (Trainings, Exercise):ti,ab,kw | 63429 |
| #11 | #6 or #7 or #8 or #9 or #10 | 189098 |
| #12 | #5 and #11 | 456 |

**
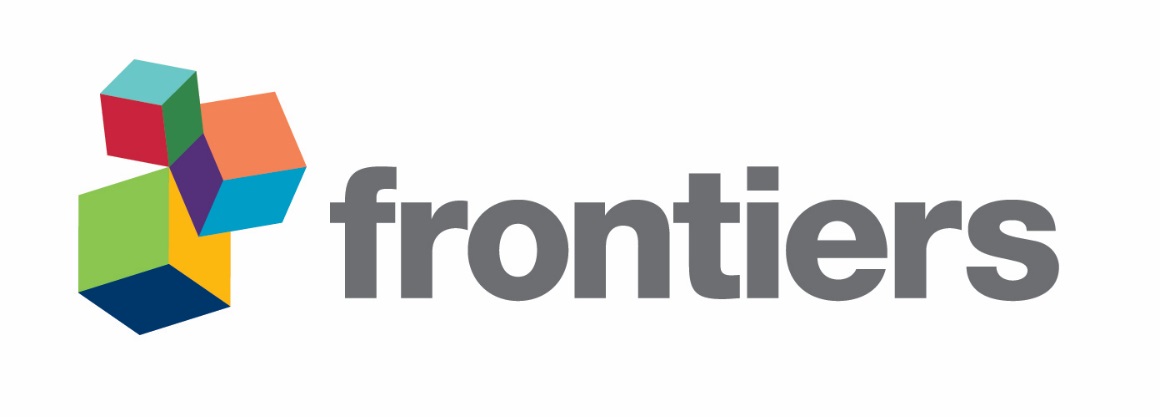
**
