# Supplementary material for: Benefits of exercise for children and adolescents with autism spectrum disorder: a systematic review and meta-analysis
Source: Front Psychiatry. 2024 Oct 7;15:1462601. doi: 10.3389/fpsyt.2024.1462601 (PMC11491325; doi:10.3389/fpsyt.2024.1462601)
Supplement: Supplementary file 2 [file Table2.docx]

Supplementary Material


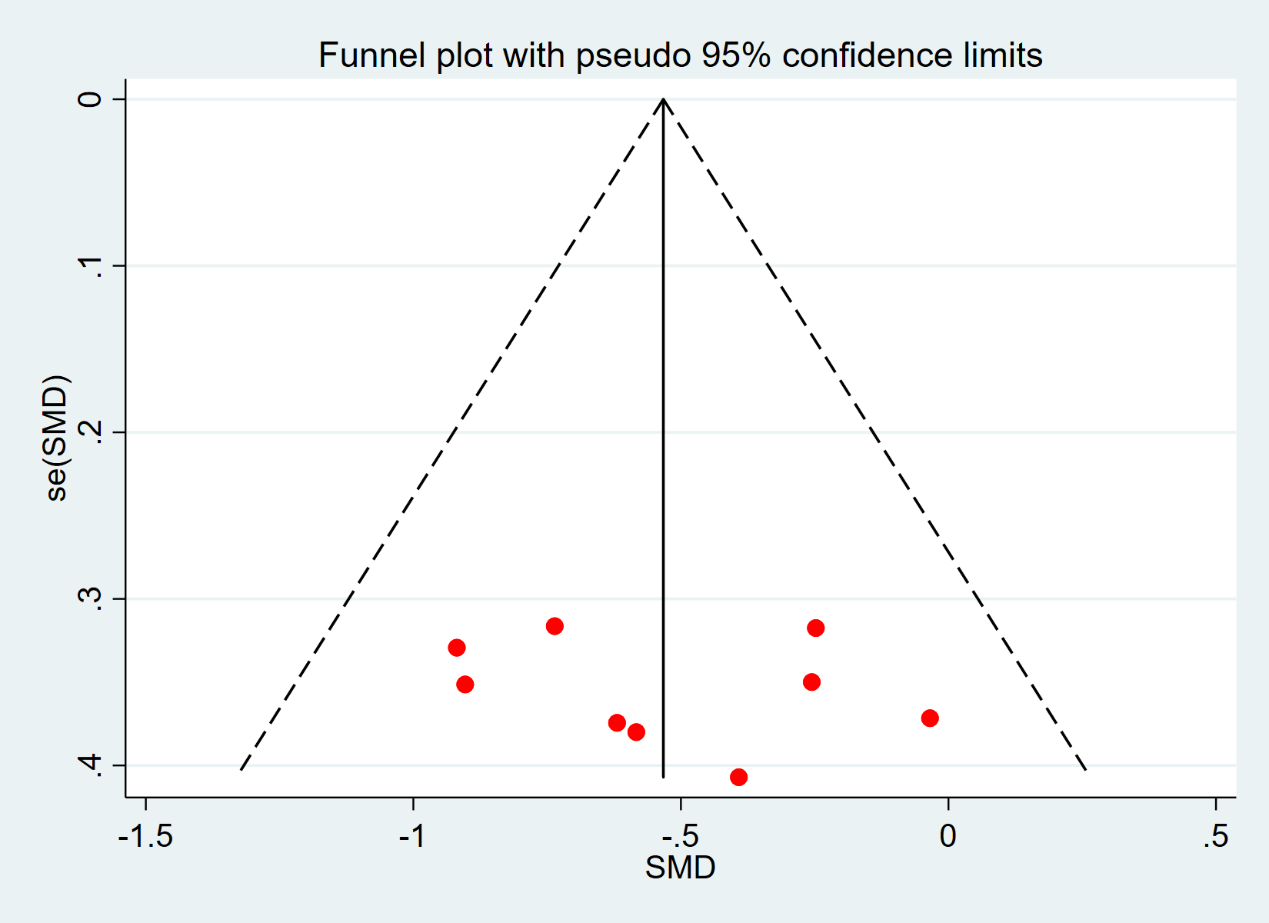


Forest plot of meta-analysis of the effect of exercise on social skills
